# A psychoacoustic test for misophonia assessment

**DOI:** 10.1038/s41598-021-90355-8

**Published:** 2021-05-26

**Authors:** Falco Enzler, Céline Loriot, Philippe Fournier, Arnaud J. Noreña

**Affiliations:** 1 Centre National de la Recherche Scientifique, Aix-Marseille University, Laboratory of Cognitive Neurosciences , 3 Place Victor Hugo, 13003 Marseille, France; 2 University of Montpellier, Montpellier, France

**Keywords:** Psychology, Human behaviour

## Abstract

Misophonia is a condition where a strong arousal response is triggered when hearing specific human generated sounds, like chewing, and/or repetitive tapping noises, like pen clicking. It is diagnosed with clinical interviews and questionnaires since no psychoacoustic tools exist to assess its presence. The present study was aimed at developing and testing a new assessment tool for misophonia. The method was inspired by an approach we have recently developed for hyperacusis. It consisted of presenting subjects (n = 253) with misophonic, pleasant, and unpleasant sounds in an online experiment. The task was to rate them on a pleasant to unpleasant visual analog scale. Subjects were labeled as misophonics (n = 78) or controls (n = 55) by using self-report questions and a misophonia questionnaire, the MisoQuest. There was a significant difference between controls and misophonics in the median global rating of misophonic sounds. On the other hand, median global rating of unpleasant, and pleasant sounds did not differ significantly. We selected a subset of the misophonic sounds to form the core discriminant sounds of misophonia (CDS_Miso_). A metric: the CDS score, was used to quantitatively measure misophonia, both with a global score and with subscores. The latter could specifically quantify aversion towards different sound sources/events, i.e., mouth, breathing/nose, throat, and repetitive sounds. A receiver operating characteristic analysis showed that the method accurately classified subjects with and without misophonia (accuracy = 91%). The present study suggests that the psychoacoustic test we have developed can be used to assess misophonia reliably and quickly.

## Introduction

Misophonia, literally hatred of sound^1^, is a condition where subjects experience negative emotional reactions (e.g., irritation, anger and/or disgust)^2,3^ and a strong autonomic arousal response when hearing specific “trigger” sounds^4,5^. These triggers most often contain human generated mouth sounds (e.g., chewing and slurping), breathing and nose sounds (e.g., heavy breathing and sniffing), throat sounds (e.g., swallowing and throat clearing), but also repetitive sounds of objects operated by humans (e.g., pen clicking and keyboard typing)^2–4^. In some cases, visual repetitive stimuli, like leg-rocking or finger tapping, can act as misophonic triggers, a phenomenon known as “misokinesia”^2–4^.

The physical characteristics of sounds (e.g., intensity and frequency) only partially influence the reaction of misophonics to triggers, rather it is their psychological profile, previous experience, and the context in which triggers are experienced that are the most important^1,4,6,7^. For instance, experiencing triggers when one cannot escape from the situation (e.g., plane trip) worsens negative reactions^4^. Also, eating and chewing sounds are less annoying when originating from babies or animals, as it is “not their fault” if they are generating them^4^. Similarly, an individual’s own chewing sounds do not trigger a reaction and are often used as a coping mechanism to “cancel out” incoming triggers^3,4^. Other coping mechanisms include listening to music, walking away, avoiding social situations, using earplugs/headphones, and asking the originator of the trigger to stop^2–4^.

Prevalence reports of misophonia show large variability and range from 6% to 49.1%^8–10^. These differ considerably due to the different assessment methods and criterion that were used to define misophonia. Besides, misophonia severity varies: Naylor et al.^10^ found that 37%, 12% and 0.3% of medical students had mild, moderate, and severe symptoms, respectively. They suggested that misophonia affects many people mildly, but only a few severely.

Misophonia can be accompanied by different comorbidities such as obsessive–compulsive personality traits, depression, and anxiety^2,3,8,11^. Perfectionism^2^, neuroticism^2,3,12^, difficulties with emotion regulation^12^, and high interoceptive sensibility^5^ are also observed. Generally, no audiological problems are detected (e.g., audiogram, loudness discomfort levels, and speech audiometry)^2,3^, and cases of tinnitus and hyperacusis are scarce (2% and 1%, respectively)^2^. Misophonia has a significant impact on the quality of life^2,8,9^ and causes daily stress because of anticipation of encounters with misophonic triggers^3^.

An fMRI study showed greater activation of the anterior insular cortex (AIC) in misophonics compared to controls when presented with trigger sounds^5^. The AIC is involved in the “salience network” which is critical in interoceptive signals and emotion processing (including anger). Increased functional connectivity of the AIC with core parts of the DMN (Default Mode Network), hippocampus, and amygdala, were also found in response to trigger sounds^5^. Two later trigger-exposure fMRI studies further supported involvement of the “salience network” in misophonia^13,14^. One of them also showed an increased synchronization of the premotor, mid-cingulate, and orbitofrontal cortices in subjects with misophonia^14^.

Misophonia is most commonly diagnosed through clinical psychological interviews and/or with questionnaires^15^. Schröder et al.^3^ described what criteria should be present to diagnose misophonia and they suggested the A-MISO-S questionnaire to assess them. These have later been reviewed by the same group^2^, and form the basis of the revised version of the A-MISO-S: the AMISOS-R. Diagnostic criteria include: Feelings of irritation, anger, and/or disgust towards specific oral or nasal human generated sounds, loss of self-control (due to impulsive physical reactions), avoidance behaviors, significant impact on the quality of life, and indications that these behaviors are not better explained by other disorders (e.g., autism or attention deficit hyperactivity disorder). Other questionnaires include the Misophonia Questionnaire^8^ and the MisoQuest^16^. The latter is the only one that has been fully validated. To date, none of these have been translated and validated in French.

To our knowledge, no psychoacoustic test exists for misophonia. While questionnaires’ performance in diagnosing misophonia and its associated distress is reasonably good, they are based on the subjects’ recollection of their experience when faced with misophonic sounds. The true lived experience of the misophonic sounds is missing. We believe that measuring this subjective experience is important in estimating the aversion of subjects when faced with triggers. Such a test would also reveal for what kind of sounds a patient presents strong feelings and/or reactions. Moreover, estimates of the unpleasantness of misophonic sounds could be used as in situ outcome measures when evaluating potential misophonia treatments.

In a previous study^17^, we designed a psychoacoustic test for the diagnosis of hyperacusis by using the ratings of natural sounds on a pleasant to unpleasant VAS (Visual Analog Scale). In this study, we propose using a similar approach but adapted to misophonia using misophonic sounds. The task was completed online by 253 subjects. Our goals were to: (i) Identify which trigger sounds were rated as most unpleasant, (ii) select an optimal subset of these to create a new assessment tool for misophonia, and (iii) further validate the use of ratings of natural sounds as a novel approach for sound-based pathologies assessment and diagnosis.

## Methods

### Subjects

Subjects were recruited through mailing lists and social networks. Notable Facebook groups were “Misophonie/Misokinésie—1er groupe de France”, “Misophonie/Misokinésie—2ème groupe de France”, “Misophonia without Borders”, “Misophonia Treatment and Management”, “Misophonia International Support Group”, “Western and Pacific Misophonia Support Group”, “CAA tinnitus and hyperacusis Special Interest group”, and “Orthophonie et Audiologie Québec”. 253 subjects took part in the study (median age = 33 years; Median Absolute Deviation (MAD) = 7 years). The only inclusion criterion was to be at least 18 years old.

The ethics committee of Aix-Marseille University approved this study (reference number: 2020-10-08-001). The study was performed in accordance with institutional guidelines and the Declaration of Helsinki, and complied with national regulations. Informed consent was obtained from all participants.

### Online task

The first page included a detailed description of the task, our contact information, and a request to do the experiment in a calm environment. No identifying information was collected, only the age. A list of questions, with explanations (shown here in brackets), was asked: (1) *Do you have hearing issues (you ask others to repeat, you have problems understanding speech in noise)?* (2) *Do you have tinnitus (ear whistling)?* (3) *Do you have auditory hypersensitivity (are some sounds loud or painful at modest intensities for you when they do not cause any reaction in others)?* (4) *How disabled are you by this hypersensitivity?* (5) *Are there any particular sounds that trigger very intense reactions in you such as anger, disgust…?* Those that responded “yes” to the last question, were asked to name what sounds trigger these reactions. Explanations could be seen by hovering the mouse over an information bubble. Questions 1, 2, 3, and 5 could be answered with “yes”, “no”, or “I don’t know”. Question 4 could be answered with “not at all”, “a little”, “moderately”, or “a lot”. For the remaining of the paper, questions 1, 2, 3, and 5 will be referred to as self-report questions for hearing issues, tinnitus, hyperacusis, and misophonia, respectively. Caution should be taken when examining our results on the self-report of hyperacusis. Reasons for this will be addressed in the discussion.

Subjects were requested to complete the MisoQuest^16^. We chose this questionnaire as it is, to the best of our knowledge, the only fully validated misophonia questionnaire. It contains 14 items. Each item is given a score from 1 to 5: (1) I completely disagree, (2) I disagree, (3) Neither agree nor disagree, (4) I agree, (5) I completely agree. The total score is obtained by summing the scores for each item, it ranges from 14 to 70. A total score above (or equal to) 61 suggests misophonia diagnosis^18^. Siepsiak et al.^18^ chose this cut-off by subtracting one standard deviation (SD = 4.3) from the mean total score of misophonics (mean = 65.72). To verify this cut-off, we performed a Receiver Operating Characteristic (ROC)^19^ analysis of data from Siepsiak et al.^16^ (Supplementary Data “raw_data2.csv”) and found that a cut-off of 61 was optimal (highest overall classification accuracy) in separating MisoQuest total scores of controls (n = 254) from scores of misophonics (n = 61).

Subjects were first presented with white noise and were asked to adjust a volume slider until sound was at a comfortable listening level. Subjects then trained with the rating of test sounds that are not part of the experimental sounds (“marimba” and “squeaking door”). This was also the opportunity to readjust the volume if necessary. Once the training phase finished, subjects were asked not to change their system sound level for the remainder of the experiment. All subsequent sounds were thus presented at individual comfortable levels.

Twenty-eight sounds were repeated three times at random and each sound had to be assessed on a VAS ranging from "very pleasant" (far left) to "very unpleasant" (far right). The words "very pleasant" and "very unpleasant" were coloured respectively in green and red to avoid any confusion. Subjects were instructed that sounds are not necessarily very pleasant or very unpleasant and that the pleasantness/unpleasantness of sounds are variable. As such, they were requested to use the full length of the scale. If the sounds were neither pleasant nor unpleasant, then the subject was instructed to respond in the middle of the scale (“neutral”). Subjects could replay the sound as many times as necessary before finalizing the answer with a button.

The test was available in English or in French and was accessible via a computer or a smartphone. It took about 20 min to complete. The MisoQuest was translated into French by the authors.

### Labeling subjects

Subjects were labeled as misophonics (n = 78; median age = 32, MAD = 8) if they self-reported misophonia and if their MisoQuest score was above (or equal to) 61. Subjects were labeled as controls (n = 55; median age = 33, MAD = 5) if they had a MisoQuest score below 61, did not self-report hearing issues, tinnitus, hyperacusis and misophonia (answered “no” or “I don’t know”), and indicated no impact of hyperacusis on their lives (answered “not at all”).

### Sounds

Sixteen misophonic sounds were selected based on previous reports of misophonia triggers in the literature^2,3^. We selected mouth, breathing, nasal, and throat sounds as well as repetitive sounds (keyboard typing and pen clicking). Six unpleasant and six pleasant sounds were also selected. These were rated as such by controls in a previous study^17^.

Sound files were retrieved from publications^17,20^, and from online sources. These are detailed in Table 1. Sound duration ranged from 0.9 s to 2.8 s (mean = 2.1 s, SD = 0.4 s). All sounds had the same root-mean-square sound pressure.

**Table 1:** List of sounds.

Misophonic trigger sounds		Unpleasant sounds	Pleasant sounds
Blowing nose $^{FrS}$	Pen clicking $^{FS}$	Clapping $^{E}$	Birds $^{E}$
Breath Running $^{F}$	Slurping $^{E}$	Distorted Guitar Dissonance $^{E}$	Fountain $^{E}$
Chewing 1 $^{E}$	Sniffing $^{FrS}$	Fingernails on Chalkboard $^{E}$	Harp $^{E}$
Chewing 2	Snoring $^{FrS}$	Fork Scratch Plate $^{E}$	Lake $^{E}$
Cough $^{FrS}$	Swallowing $^{Y}$	Knife Hit Glass $^{E}$	Laugh $^{E}$
Gargling $^{FS}$	Throat Clearing $^{FS}$	Scream $^{E}$	Underwater $^{E}$
Hard Breathing $^{FrS}$	Vomit $^{FrS}$		
Keyboard $^{E}$	Wheezing $^{FrS}$		

^*E*,^^*F*,^^*FS*,^^*FrS*^ and ^*Y*^ indicate where sounds were retrieved: Enzler et al.^17^, Fan et al.^20^, www.fesliyanstudios.com, www.freesound.org, and www.youtube.com, respectively. “Chewing 2” was self-created and recorded in the same session than sounds from Enzler et al.^17^, but it was not used in the final list of that publication.

### Statistical analysis

Sound ratings’ analysis was first attempted with a linear mixed-effects model. However, the distribution of its residuals did not fulfill assumptions of linearity or homoscedasticity. Together with observations of sound ratings’ distributions, it was clear that our data did not show gaussian distributions (this was also confirmed with Lilliefors tests). We thus used non-parametric tests. When testing the null hypothesis that two samples were equal, we used the Wilcoxon non-parametric two-tailed rank test. When multiple comparisons were performed, α was corrected with the Bonferroni correction. Intraclass Correlation Coefficient (ICC) estimates and their 95% confidence intervals were calculated based on a mean-rating (across repetitions, k = 3), absolute agreement, 2-way mixed-effects model (i.e. ICC(2,3)). ICC values range between 0 and 1. Values below 0.5, between 0.5 and 0.75, between 0.75 and 0.9, and above 0.9 indicate poor, moderate, good, and excellent reliability, respectively^21^. Statements on ICC reliability were made with respect to 95% confidence intervals. To compute effect sizes between median sound ratings for two groups, we used the *r* effect size^22^. Values of *r* near 0.5, 0.3 or 0.1 indicate, respectively, large, medium, and small effect sizes. To compare the performance of classifiers, we used ROC curves^19^. These allow comparison of specificity (proportion of negatives (e.g., controls) correctly identified as negatives) and sensitivity (proportion of positives (e.g., misophonics) correctly identified as positives) of classifiers for different cut-off values. The higher the AUC (Area Under Curve) of these curves, the better the classifier. The maximum possible AUC is 1, which would indicate a perfect classifier. The Wilson score with continuity correction^23^ was used to estimate 95% confidence intervals for accuracy, specificity, and sensitivity results. For correlations, Spearman’s ρ (rho) was used. To test the effect of repetition on sound ratings, we built a zero–one inflated beta model using R (R Core Team 2021) and the brms package^24,25^.All other above computations and following figures were done with MATLAB 2019a.

## Results

### Questionnaire data

Frequency of answers to self-report questions one to five and how they compare to each other are shown in Supplementary Table S1.

**Figure 1:**
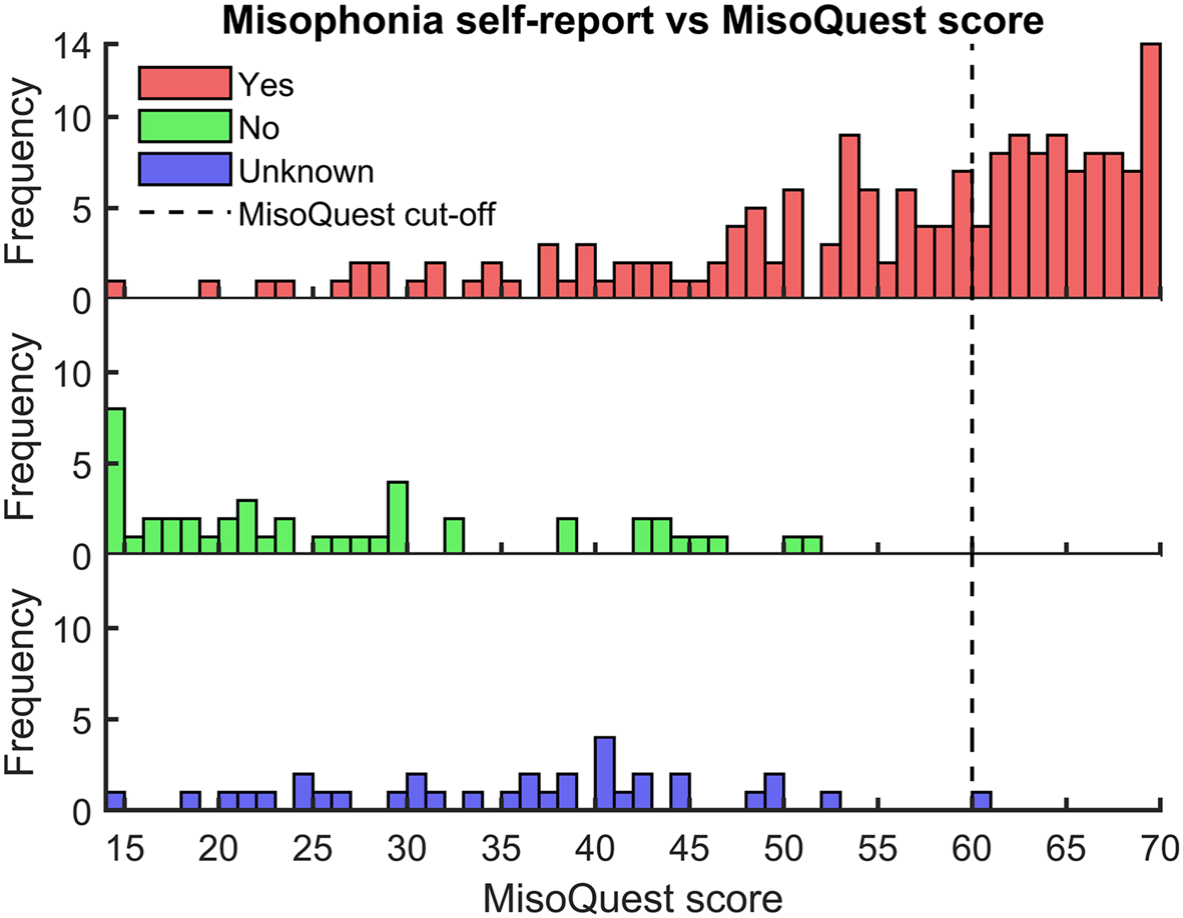
Misophonia self-report vs MisoQuest score histograms. Frequency of MisoQuest scores for subjects answering “yes”, “no”, and “I don’t know” to self-report of misophonia are shown in red, green, and blue, respectively. The MisoQuest diagnostic cut-off (= 61) is shown with a black dotted line. Histogram bins contain the upper values of bin edges (e.g., the subject with the highest score in the bottom plot had a value of 61), as such the cut-off is drawn at x = 60.

69% of recruited subjects self-reported misophonia. These had varying degrees of misophonia severity. As shown in Fig. 1, their MisoQuest scores ranged from 14 to 70. The MisoQuest cut-off value was highly specific to the self-report of misophonia: 99% of subjects not self-reporting misophonia (or being unsure) had a MisoQuest score below 61. On the other hand, it was not very sensitive: 45% of subjects with a self-report of misophonia had a MisoQuest score above (or equal to) 61.

Frequencies of diagnoses with the MisoQuest and the self-report question on misophonia are shown with respect to other self-report questions (hearing issues, tinnitus, hyperacusis, and hyperacusis impact) in Table 2. 22%, 19%, and 71% of misophonics self-reported hearing issues, tinnitus, and hyperacusis, respectively.

**Table 2:** Frequency of misophonia diagnoses using the MisoQuest and self-report of misophonia and how they compare to self-report of hearing issues, tinnitus, hyperacusis, and hyperacusis impact.

MisoQuest > = 61?	Misophonia?	N	Hearing Issues?			Tinnitus?			Hyperacusis?			Hyperacusis Impact?			
			Yes	No	Un	Yes	No	Un	Yes	No	Un	A Lot	Mod	A Little	Not At All
**Yes**	**Yes**	**78**	**17**	**50**	**11**	**14**	**55**	**9**	**55**	**17**	**6**	**34**	**17**	**8**	**19**
Yes	No	0	0	0	0	0	0	0	0	0	0	0	0	0	0
Yes	Unknown	1	0	0	1	0	0	1	0	0	1	0	0	0	1
No	Yes	96	10	76	10	13	79	4	48	37	11	6	21	24	45
No	No	45	5	**﻿38**	**﻿2**	8	**﻿36**	**﻿1**	8	**﻿36**	**﻿1**	0	2	5	**﻿38**
No	Unknown	33	4	**26**	**﻿3**	4	**﻿26**	**﻿3**	3	**﻿24**	**﻿6**	0	0	4	**﻿29**
	N	253	36	190	27	39	196	18	114	114	25	40	40	41	132
	*Controls*	*55*	*0*	*51*	*4*	*0*	*52*	*3*	*0*	*51*	*4*	*0*	*0*	*0*	*55*

“Misophonia?”, “Hearing Issues?”, “Tinnitus?”, “Hyperacusis?”, and “Hyperacusis Impact?” refer to questions 5, 1, 2, 3, and 4, respectively. Subjects were labeled as misophonics if they had a MisoQuest score above (or equal to) 61, and if they self-reported misophonia (bold). Subjects were labeled as controls if they had a MisoQuest score below 61, if they answered “no” or “unknown” to questions 1, 2, 3, and 5, and if they answered “not at all” to question 4 (﻿italics).

*Un.* unknown = “I don’t know”, *Mod.* moderately.

Median MisoQuest scores for misophonics and controls were 65 (MAD = 2; range: 61—70) and 27 (MAD = 9; range: 14—52), respectively.

**Figure 2:**
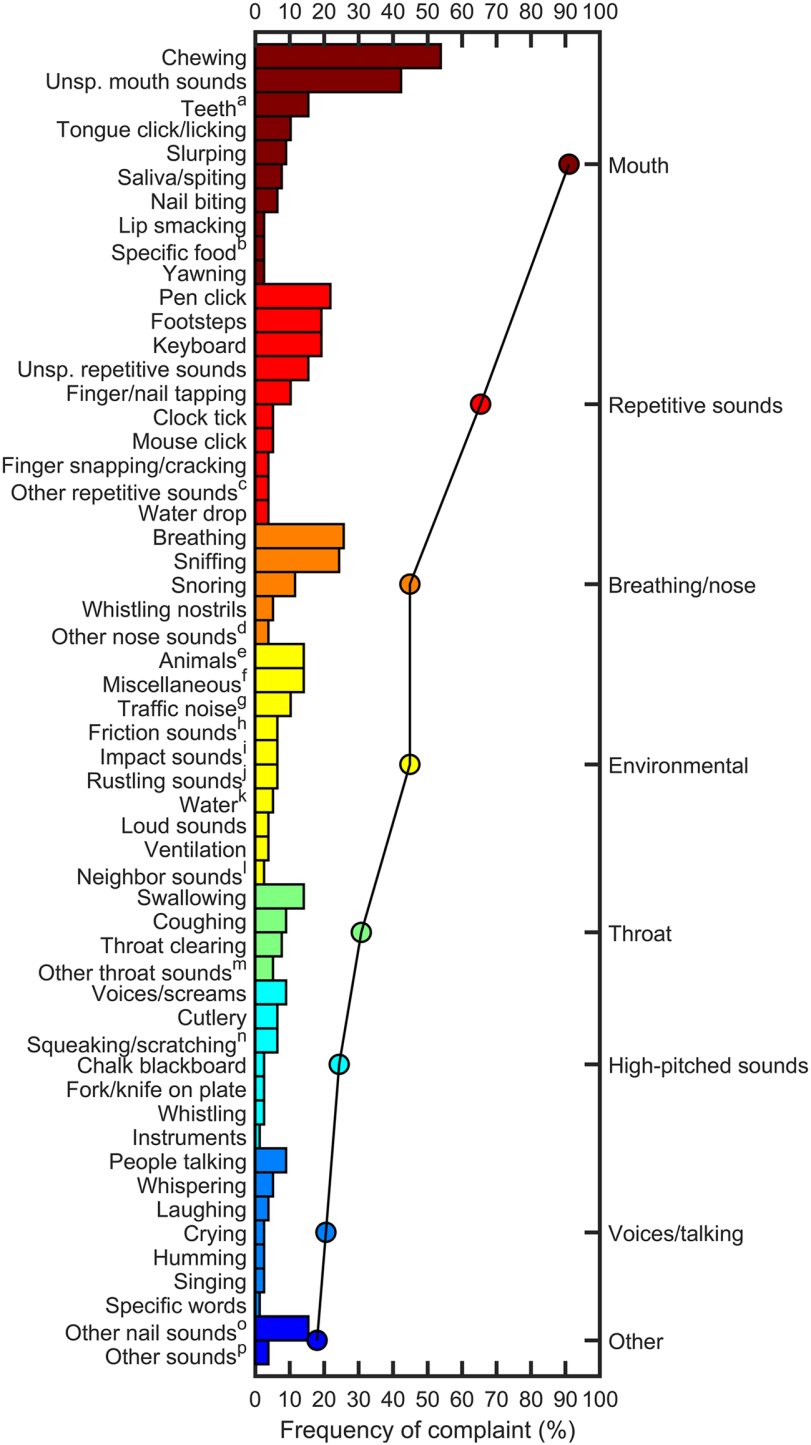
Sounds reported by misophonics as triggers. The percentage of misophonics that reported a given sound, shown on the left y axis, as a trigger are indicated with bars. Each sound was assigned to one of eight categories (right y axis). Sounds are colored with respect to their assigned category. Percentages of misophonics for each category (round symbols), represent how many misophonics reported at least one sound within that category as a trigger. Misophonics could report more than one sound within each category. *Unsp.* Unspecified, which indicates that no specific sound was named, for instance: “any repetitive sound”. ^a^Brushing, friction, sucking, fork hitting. ^b^Apple bite, popcorn, chips. ^c^Music, motor, words (“um”, “like”). ^d^Sneezing, snorting, unspecified. ^e^Dog barking, rooster, cats, birds. ^f^Plane, church bell, keys, electronic cigarette, bones cracking, construction work, heater, washing hands, writing on table, sliding window, belt buckle, football, clapping, crowded party. ^g^Car, motorbike, horn, siren. ^h^Clothes, headphones, hands, scuffing shoes. ^I^ Door slamming, cymbal. ^j^Paper compaction, turning pages, bag of chips. ^k^Leak, running, rain. ^l^Television, music. ^m^Burping, gagging, gurgling, unspecified. ^n^Breaks, guitar strings, metal scratching, windscreen wipers, door. ^o^Clipping, filing, scratching, snapping, unspecified. ^p^Thumping, leg shaking, tinnitus.

Answers by misophonics on the open question of what sounds they considered as triggers are shown in Fig. 2. Sounds were arbitrarily separated in categories. Some sounds were assigned to repetitive or high-pitched sounds because subjects specified it: for instance, “repetitive music”, or “high-pitched voices”. Some subjects reported several sounds within the same category. The most common trigger type reported by misophonics was mouth sounds: 91% reported at least one type of mouth sound, with 54% naming specifically chewing sounds, and 42% reporting general mouth sounds. 65% reported at least one type of repetitive sound, with 22%, 19%, and 19%, naming specifically pen click, keyboard typing, and footsteps, respectively. 45% and 31% reported at least one type of breathing/nose, and throat sound, respectively. Detailed values for each sound and category are shown in Supplementary Table S2.

Some responses were very specific, for instance: “bare feet walking on floors covered with a plastic layer, which creates a sort of suction sound”, “headphone friction on my ears”, “voice of my partner when singing”, and “repetitive words such as “um” and “like””.

Some sounds might be more associated to symptoms of hyperacusis than of misophonia, for instance loud traffic noises or high-pitched sounds^26,27^. Only one subject mentioned hyperacusis as a reason for the presence of two sounds: birds, and cymbal. This subject also reported high-pitched sounds like screams, squeaking sounds, and instruments (e.g., violin). (S)he did not specify if the latter were due to hyperacusis or misophonia.

### Sound ratings

The VAS positions were mapped to ratings that went from 0 (highly pleasant) to 100 (highly unpleasant), where 50 was neither pleasant nor unpleasant.

For each subject and sound, we computed the standard deviation (SD) of ratings across three repetitions. For each subject, we averaged SDs across all sounds. This gives a general measure of how reliable (mean of SDs) each subject is across repetitions (low values indicate good reliability). The mean and SD of these values were 5.7 and 2.4, respectively. Subjects with their mean SDs above 10.8 (mean + 2SD) were excluded (n = 9). Three had been labeled as misophonics and one had been labeled as control. Following results on sound ratings are therefore based on 75 misophonics (median age = 32, MAD = 8) and 54 controls (median age = 33, MAD = 5).

To test the effect of repetition on sound ratings (from misophonics and controls with reliable ratings), we built a zero–one inflated beta model with group and repetition as fixed effects, and subject and sound as random effects. For the mean of ratings, the model revealed a significant effect of group (95% Credible Interval (CrI): [0.033, 0.085]), but not of repetition (95% CrI for 2nd vs 1st, 3rd vs 2nd, and 3rd vs 2nd repetitions were, respectively, [-0.0064, 0.0092], [-0.0015, 0.014], and [-0.0015, 0.014]). For each subject and sound, we thus averaged ratings across their three repetitions.

ICCs(2,3) were computed for each sound (for subjects with reliable ratings) and showed good to excellent reliability between repetitions. Mean ICC(2,3) across all sounds was 0.93. 16 sounds (out of 28) had excellent reliability. Full ICC(2,3) details are shown in Supplementary Table S3.

**Figure 3:**
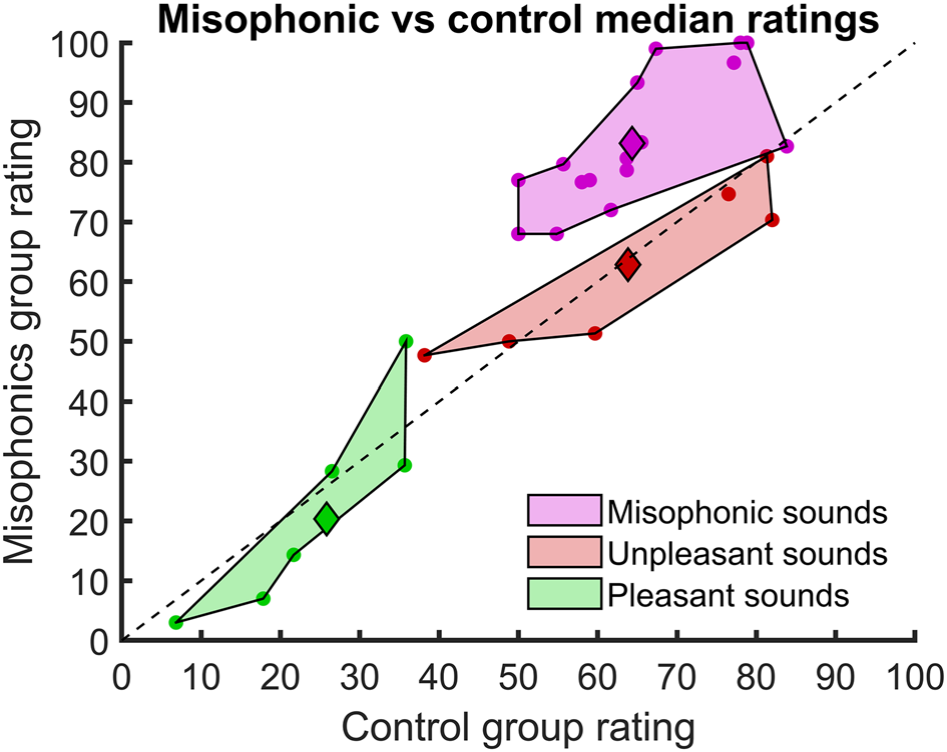
Misophonic vs Control median ratings for each sound. Ratings vary between 0 (Pleasant) and 100 (Unpleasant). Each dot represents the median rating of a sound for the control (x-axis) group and the misophonic (y-axis) group. Sounds are colored and grouped as misophonic (magenta), unpleasant (red), and pleasant (green), as determined initially during the choice of the stimuli (Table 1). The median of individual medians of sound ratings within the same category is shown as a diamond. As a visual reference, the $y=x$ line is drawn in dotted black. Sounds close to this line have similar ratings for the two groups.

Figure 3 compares the median ratings of each sound for the controls and misophonics. For each subject, we computed the median of their sound ratings for each category. The median for each sound category was then calculated across subjects for each group (control and misophonic). The difference of medians between the misophonic and control groups (i.e., y–x coordinates of diamond symbols in Fig. 3) was markedly larger for misophonic sounds (difference = 18.3) than for unpleasant (difference = − 1) or pleasant sounds (difference = − 5.5). Rank tests were used to evaluate the null hypothesis that median ratings for sounds with the same category were the same for the two groups. Misophonic sounds’ medians were significantly different between both groups (p = $5.3 \cdot {10}^{-9}$; r = 0.51). Unpleasant and pleasant sounds’ medians did not show significant differences (p = 0.75, and p = 0.50, respectively; r = − 0.03, and − 0.06, respectively).

**Figure 4:**
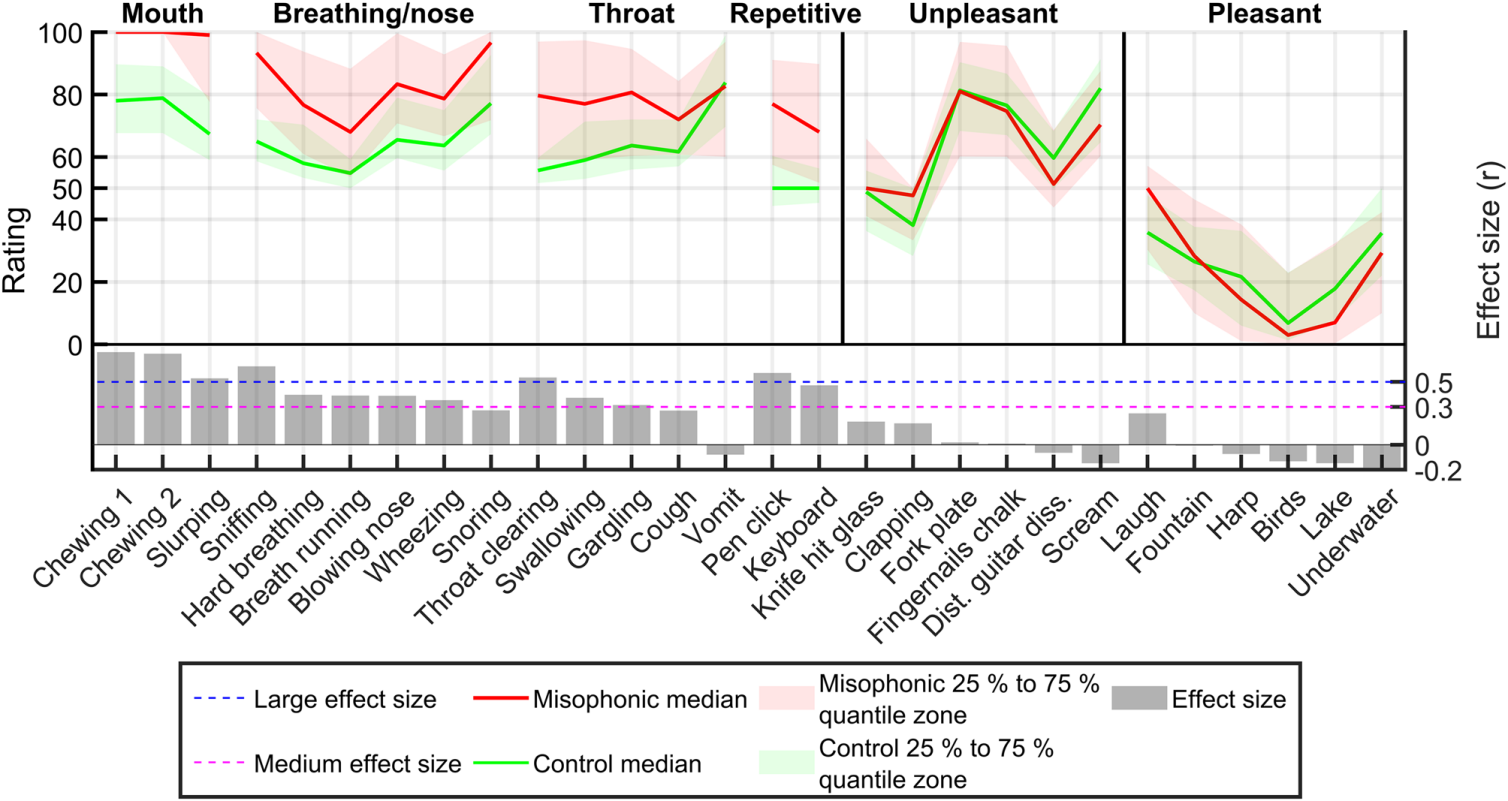
Ratings and effect sizes for each sound. Each sound is shown on the x axis. They are ordered by category (misophonic, unpleasant, and pleasant), subcategory for the misophonic sounds (mouth, breathing/nose, throat, and repetitive), and by decreasing effect size within each (sub)category. The left y axis represents sound ratings, which vary between 0 (highly pleasant) and 100 (highly unpleasant). Control and misophonic medians and 25% to 75% quantiles, are indicated in green and red, respectively. The right y axis represents the effect size (r). Large and medium effect sizes (r = 0.5 and 0.3) are shown as blue and magenta horizontal lines, respectively. Each sound’s effect size is shown as a grey bar plot. *Dist.* Distorted, *diss.* dissonance.

Misophonic sounds were separated in four subcategories based on categories described in the literature^2,3^, and those observed in Fig. 2: mouth, breathing/nose, throat and repetitive sounds. Figure 4 shows the ratings of controls and misophonics, and the effect size, for each sound. The effect size allows us to determine the most discriminant sounds, i.e., those that can best separate control from misophonic ratings. In accordance with Fig. 3, misophonic sounds had the highest effect sizes, while unpleasant and pleasant sounds had low effect sizes. Mouth and repetitive sounds had the highest mean effect size: 0.66 and 0.52, respectively. “Chewing 1”, “Chewing 2”, “Slurping”, “Sniffing”, “Throat Clearing”, and “Pen Click”, had large effect sizes (r > 0.5): 0.74, 0.73, 0.53, 0.62, and 0.57, respectively. “Vomit” had a low effect size (− 0.08) and was rated as very unpleasant for both groups. This suggests that it was wrongfully categorized as a misophonic trigger sound.

### Core discriminant sounds

To create a new assessment tool, we wanted to select the sounds with the most discriminative power whilst keeping in mind clinical practicalities. This tool should not be too time-consuming (not too many sounds), and it should capture the main complaints of misophonics. As shown in Fig. 2, several categories of sounds were considered as triggers. Moreover, some subjects would report mouth sounds as triggers, and not mention any repetitive sounds. The contrary was seen too. Hence, the final choice of sounds should assess these putative dimensions of misophonia. As in Enzler et al.^17^ for hyperacusis, the optimal subset of sounds to assess misophonia were called Core Discriminant Sounds (CDS). For clarity, the first will be referred to as CDS_Hyp_ and the latter as CDS_Miso_.

As seen in Figs. 3 and 4, misophonic sounds discriminate best misophonic from control ratings. As such, the CDS_Miso_ were selected within these. We wanted to keep sounds from each subcategory of misophonic sounds, to assess the unpleasantness of each in the CDS_Miso_. To identify what sounds were the most important in discriminating controls from misophonics in each subcategory, we first defined a metric that could measure this: the CDS score. Second, we computed this metric for different choices of CDS and compared their performance using ROC curves^19^.

To compute the CDS score, we took an approach similar to the definition of dB HL, where 0 dB HL is defined as a normalized value i.e., a value that represents the behavior of a control population. Positive values are deemed different than normal when crossing a chosen threshold (usually 20 dB HL). Our goal was to create a metric that evaluates how different subjects’ ratings are from a given threshold. We set a threshold for each sound at the 75% quantile of the control group’s distribution. For a given subject, a given sound ($s \in CDS=\left\{\mathrm{1,2},3,\dots , |CDS|\right\}$ where each index represents one of the $CDS$ and $|CDS|$ is the number of elements within this set), we compute the distance, in percentage, of this sound’s rating (${Rating}_{s}$) from its respective 75% quantile (${Quantile}_{.75,s}$):

$${Distance}_{s}= \frac{{Rating}_{s}- {Quantile}_{.75,s}}{100- {Quantile}_{.75,s}} \cdot 100$$

with ${Rating}_{s}- {Quantile}_{.75,s}=0$ if ${Rating}_{s}< {Quantile}_{.75,s}$ (we only want to evaluate positive differences) and ${Distance}_{s}= 0$ if ${Quantile}_{.75,s}=100$ (to avoid division by 0). $100- {Quantile}_{.75,s}$ is the maximum possible distance of a rating from its respective quantile. The CDS score expresses how high a given subject’s ratings are relative to the quantiles. For a given set of sounds (the CDS), the CDS score is computed by averaging the distances of sound ratings within that set and whose quantiles are below 100. This subset of sounds is defined as: $s\_valid=\left\{s \in CDS \right| {Quantile}_{.75,s}<100\}$. Indeed, for sounds with a 75% quantile (${Quantile}_{.75,s}$) equal to 100, their ${Distance}_{s}$ becomes zero. However, we do not want to include this null distance in our average. In this study, no sound had their 75% quantile of control ratings equal to 100. The CDS score is defined as:

$$CDS \, Score =\frac{1}{|s\_valid|}\sum_{x \, \in \, s\_valid} \, {Distance}_{x}$$

where $\left|s\_valid\right|$ is the number of sounds in the CDS with a 75% quantile below 100.

For each CDS score, we computed the cut-off value that best separates misophonic scores from control scores i.e. the value where scores above (or equal to) this cut-off indicate misophonia and scores below this cut-off indicate no misophonia. The cut-off values were obtained by maximizing classification accuracy. Accuracy was calculated by dividing the sum of true positives (misophonics with a score above (or equal to) the cut-off) and true negatives (controls with a score below the cut-off) by the total number of subjects (75 misophonics + 54 controls = 129 subjects).

For each subcategory, we tested every combination of k sounds possible, with k ranging from 1 to *n*, where n is the number of sounds within that subcategory. In other words, we tested CDS that contained 1 to *n* sounds, and for each number of sounds (k), we tested all the possible ways we could select k sounds from *n* sounds.

**Figure 5:**
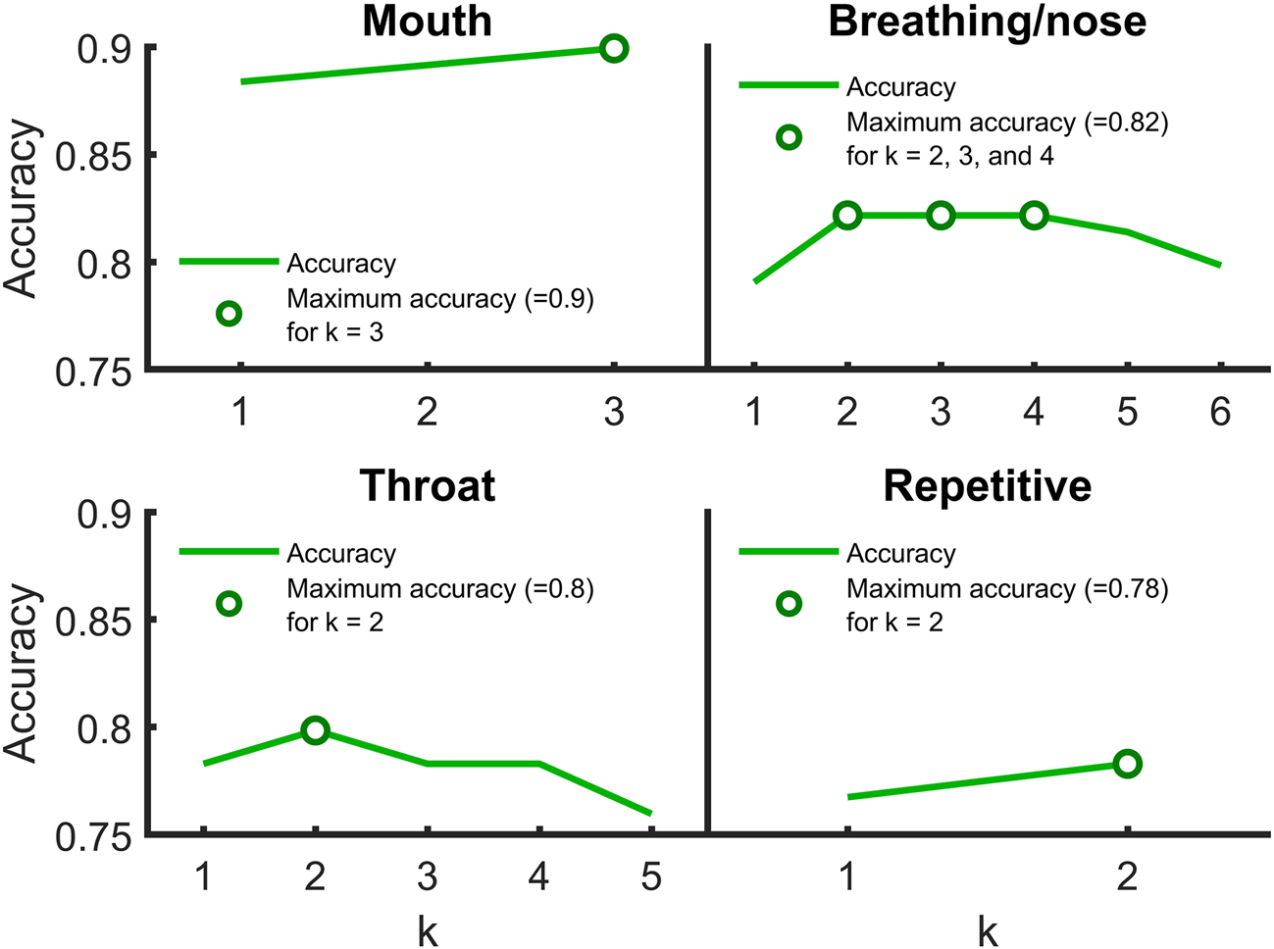
Performance of CDS score for the best combination of CDS for each k within each subcategory. The highest accuracy for each k is shown by a green line. Maximum accuracy(ies) for each subcategory is(are) shown by round green symbols.

For each k, we selected the combinations of CDS that gave the highest accuracy. Figure 5 shows the highest accuracy, for each k, and for each subcategory. The best subset of sounds for mouth sounds, CDS_Mouth_, were “Chewing 1”, “Chewing 2”, and “Slurping”. For breathing/nose sounds, maximum accuracy was the same for k = 2, 3, and 4. Maximum AUC for these were 0.870, 0.873, and 0.860, respectively. As such, the most accurate combination of three sounds was chosen for CDS_Breathing/Nose_. They contained one breathing sound and two nose sounds: “Breath Running”, “Sniffing”, and “Snoring”. The CDS_Repetitive_ were “Pen Click”, and “Keyboard”. The CDS_Throat_ were “Throat Clearing”, and “Swallowing”. These sounds form the CDS_Miso_ (n = 10).

**Figure 6:**
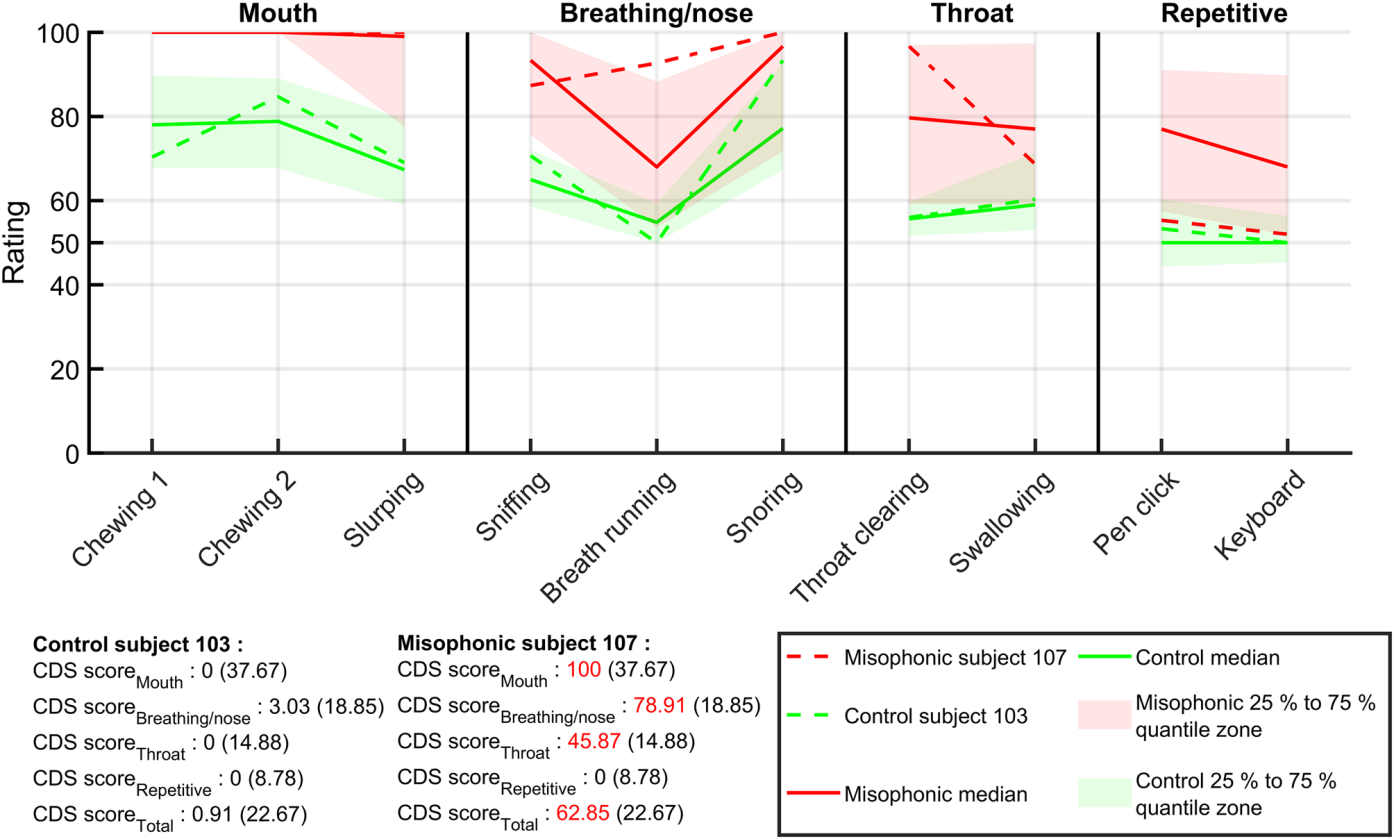
CDS_Miso_ ratings. Each CDS is shown on the x axis. They are ordered by subcategory (mouth, breathing/nose, throat, and repetitive), and by decreasing effect size within each subcategory. The left y axis represents sound ratings, which vary between 0 (highly pleasant) and 100 (highly unpleasant). Control and misophonic medians and 25% to 75% quantiles, are indicated in green and red, respectively. Control and misophonic individual examples are indicated by dotted lines. The CDS scores are colored in red if they are above (or equal to) respective cut-off values (in brackets).

Ratings of the CDS_Miso_ for both groups are shown in Fig. 6. Two example subjects (one control and one misophonic) are also shown with their respective CDS scores. For each subcategory, we computed their CDS score. We also computed a global score, ${CDS \, Score }_{Total}$, which is computed by including all sounds from the CDS_Miso_. Optimal cut-off values, computed with ROC analysis, are indicated next to each score in brackets. Hence, for each score, we can assess if subject’s ratings are abnormal. For instance, a ${CDS \, Score }_{Mouth}$ above (or equal to) 37.67 suggests that a subject was abnormally annoyed by mouth sounds. More generally, a ${CDS \, Score }_{Total}$ above (or equal to) 22.67 suggests misophonia. Scores for misophonic subject 107 were above respective cut-off values for ${CDS \, Score }_{Mouth},$ ${CDS \, Score }_{Breathing/Nose}$, ${CDS \, Score }_{Throat}$, and ${CDS \, Score }_{Total}.$ This suggests that this subject’s misophonia was specific to mouth, breathing/nose, and throat sounds, but not to repetitive sounds. Subject 107 was 40 years old and self-reported misophonia and hyperacusis, the latter with no impact on her/his life. (S)he did not self-report tinnitus or hearing issues and had a MisoQuest score of 62. This subject reported chewing, breathing, and gagging as trigger sounds. Control subject 103 was 29 years old, did not self-report misophonia, hyperacusis, tinnitus and hearing issues, and had a MisoQuest score of 14.

Across all recruited subjects (with reliable sound ratings), each CDS score correlated positively with the MisoQuest score (ρ = 0.67, 0.53, 0.54, 0.53, and 0.71, for mouth, breathing/nose, throat, repetitive, and total, respectively; p = $2.1 \cdot {10}^{-33}$, $9.3 \cdot {10}^{-19}$, $8.7 \cdot {10}^{-20}$, $3.6 \cdot {10}^{-19}$, and $5.4 \cdot {10}^{-39}$, respectively). Classification performances of each CDS score in separating controls from misophonics are shown in Table 3.

**Table 3:** Classification performance of CDS scores.

CDS Score	Ac. (CI, %)	Spec. (CI, %)	Sens. (CI, %)	AUC	Cut-Off
Total	91 (85–96)	87 (80–92)	95 (89–98)	0.947	22.67
Mouth	90 (83–94)	85 (78–91)	93 (87–97)	0.923	37.67
Breathing/Nose	82 (74–88)	78 (70–85)	85 (78–91)	0.873	18.85
Throat	80 (72–86)	83 (76–89)	77 (69–84)	0.849	14.88
Repetitive	78 (70–85)	83 (75–89)	75 (66–82)	0.819	8.78

CDS scores are ordered by decreasing Accuracy. Accuracy (Ac.), Specificity (Spec.), and Sensitivity (Sens.) are computed using the optimal cut-off value obtained by ROC analysis on misophonic and control subjects.

*AUC* area under curve of ROC, *CI* 95% Confidence interval.

With each subcategory of CDS_Miso,_ we could identify different behaviors from one subject to another. For instance, for misophonic subject 107 in Fig. 6, repetitive sounds were not more unpleasant than controls, but other subcategories were. In Table 4, we identified such patterns by counting misophonic subjects that had the same subcategories’ CDS score above (or equal to) their respective cut-offs. Most misophonics (55%) had all subcategories above their respective cut-offs. 15% showed no abnormality for repetitive sounds, while they did for all other subcategories. Conversely, 3% showed abnormality for repetitive sounds, while they did not for all other subcategories.

**Table 4:** Frequency of misophonic subject’s subcategory profiles.

Above (or equal to) subcategory’s CDS Score cut-off?				N
Mouth	Breathing/nose	Throat	Repetitive	
Yes	Yes	Yes	Yes	41
Yes	Yes	Yes	No	11
Yes	Yes	No	Yes	5
Yes	Yes	No	No	6
Yes	No	Yes	Yes	4
Yes	No	No	Yes	2
Yes	No	No	No	1
No	Yes	Yes	Yes	1
No	No	Yes	Yes	1
No	No	No	Yes	2
No	No	No	No	1

“Yes” indicates that the CDS score of the given subcategory is above (or equal to) its cut-off. “No” indicates that the CDS score is below its respective cut-off.

Median ${CDS \, Score }_{Total}$ for controls and misophonics were 4.45 (MAD = 11, range: 0–58) and 55.04 (MAD = 19, range: 0–100), respectively.

Subjects with no (or unsure) self-report of misophonia (and reliable ratings, n = 76) had a ${CDS \, Score }_{Total}$ median of 5.23 (MAD = 12, range: 0–78), their ${CDS \, Scores }_{Total}$ were statistically different from misophonic ${CDS \, Scores }_{Total}$ (rank test p = $1.79 \cdot {10}^{-20}$; r = 0.75), and 85.5% of them had ${CDS \, Scores }_{Total}$ below the diagnostic cut-off (i.e. 22.67, c.f. Table 3). Conversely, subjects with a self-report of misophonia (and reliable ratings, n = 168) had a ${CDS \, Score }_{Total}$ median of 42.17 (MAD = 21, range: 0–100), their ${CDS \, Scores }_{Total}$ were statistically different from control ${CDS \, Scores }_{Total}$ (rank test p = $1.91 \cdot {10}^{-15}$; r = 0.53), and 76.8% of them had ${CDS \, Scores }_{Total}$ above the diagnostic cut-off.

Median ${CDS \, Score }_{Total}$ for subjects with (n = 33) and without (or unsure, n = 211) self-report of hearing issues (with reliable ratings) were 30.38 (MAD = 28, range: 0–99.8) and 29.23 (MAD = 22, range: 0–100), respectively. ${CDS \, Scores }_{Total}$ were not significantly different between subjects with and without (or unsure) self-report of hearing issues (with reliable ratings, rank test p = 0.164; r = 0.089).

Median ${CDS \, Score }_{Total}$ for subjects with (n = 37) and without (or unsure, n = 207) self-report of tinnitus (with reliable ratings) were 27.93 (MAD = 27, range: 0–99.8) and 29.32 (MAD = 23, range: 0–100), respectively. ${CDS \, Scores }_{Total}$ were not significantly different between subjects with and without (or unsure) self-report of tinnitus (with reliable ratings, rank test p = 0.25; r = 0.073).

Median ${CDS \, Score }_{Total}$ for subjects with (n = 108) and without (or unsure, n = 136) self-report of hyperacusis (with reliable ratings) were 46.97 (MAD = 23, range: 0–100) and 17.64 (MAD = 18, range: 0–77.9), respectively. ${CDS \, Scores }_{Total}$ were significantly different between subjects with and without (or unsure) self-report of hyperacusis (with reliable ratings, rank test p = $1.19 \cdot {10}^{-13}$; r = 0.47). This result is interesting as it suggests that subjects may confuse hyperacusis and misophonia. See the next section for a discussion on the self-report of hyperacusis..

## Discussion

### Summary of the findings

With this study we wanted to create a tool that could assess misophonia by directly confronting subjects with trigger sounds. First, we found that misophonics’ unpleasantness towards sounds was specifically higher for misophonic sounds, while general pleasant or unpleasant sounds were not different from control ratings. Second, we identified a subset of sounds, the CDS_Miso_, that could be used to assess misophonia. They could also evaluate potential subcategories of misophonia. A metric, the CDS score, was used to quantify misophonia and its subcategories.

### Questionnaire data on hearing

Previous studies^2,3^ have found audiological problems to be rare in misophonics. In our study, 22% of misophonics self-reported hearing issues. It is unclear whether our findings suggest that hearing issues are missed by clinical measures in misophonics, or if the validity of online questionnaire data is to be questioned. Jager et al.^2^ had 109 misophonics (randomly selected from 575 misophonics) perform an audiogram (air and bone conduction thresholds from 0.25 to 8 kHz, and from 0.25 to 2 kHz, respectively, both in octave steps). 97% (n = 106) had bilateral normal hearing. However, they did not perform speech discrimination measures. Schröder et al.^3^ had only five subjects (out of 42) undergo hearing tests (pure tone, speech audiometry, and loudness discomfort levels). One patient showed conductive hearing loss. The other four showed no audiological abnormalities. Findings might have been different if the remaining 37 subjects had been tested. Our question on hearing issues indicates two examples in its explanation: *“you ask others to repeat”* (eventually hearing loss), and *“you have problems understanding speech in noise”* (speech in noise issues). Given results on hearing loss by Jager et al.^2^, our results might be more representative of speech in noise issues. Still, Jager et al.^2^ did not test high frequencies (above 8 kHz).

71% of misophonics reported hyperacusis. This prevalence is much higher than the 1% found by Jager et al.^2^ or of the 25% found by Sanchez and Silva^28^. We believe the high prevalence of hyperacusis in misophonics in our study should be taken with caution. Indeed, no diagnostic measure of hyperacusis was used, only one self-report question. Most importantly, the question on hyperacusis (*“do you have auditory hypersensitivity?”*) might have been wrongfully interpreted as positive for misophonics, even though no hyperacusis was present. Explanation (i.e., definition of hyperacusis) for that question (*“are some sounds loud or painful at modest intensities for you when they do not cause any reaction in others”*) was only shown when hovering over an information bubble. Subjects might have missed this. For future online studies, better care should be taken to word this question.

19% of misophonics reported tinnitus. Our results are higher than those by Jager et al.^2^, where only 2% of misophonics had tinnitus. However, the latter had only been diagnosed as such prior to the study. It is not stated whether all subjects had undergone screening for tinnitus (and hyperacusis) prior to the study or not. On the other hand, Sanchez and Silva^28^ reported that 50% of misophonics self-reported tinnitus. Furthermore, with our data, we cannot be sure that tinnitus was chronic.

Result discrepancies indicate that a putative link between tinnitus, hyperacusis, hearing loss, and misophonia is unclear.

### The MisoQuest and misophonic sounds

Siepsiak et al.^18^ found that the MisoQuest was very specific and not very sensitive: 96% and 66%, respectively. They compared MisoQuest scores to face-to-face interview diagnosis using the diagnostic criteria by Schröder et al.^3^. We found similar results: 99% specificity and 45% sensitivity, when comparing MisoQuest scores with self-diagnosis. 55% of subjects self-diagnosing misophonia were not diagnosed as such by the MisoQuest. This highlights the putative variability in misophonia diagnosis (and severity). This echoes the rather high prevalence rates of misophonia found by Wu et al.^8^ (20%) and Naylor et al.^10^ (49.1%). The latter have stated that only 0.3% had severe cases of misophonia, and that misophonia seemed to affect many people mildly, but only a few severely. As discussed by Edelstein et al.^4^, controls and misophonics find similar sounds to be aversive, but the degree of aversion experienced by misophonics is higher, which is in broad agreement with our findings (Fig. 4). The line between slightly abnormal annoyance towards specific sounds and its significant impact on the quality of life is what makes misophonia a serious condition.

91%, 65%, 44%, and 31% of misophonics reported at least one mouth, repetitive, breathing/nose, and throat sound, respectively, as a trigger (Fig. 2). These are similar proportions than those shown by Jager et al.^2^ : 96%, 74%, 85%, and 69% for eating, repetitive tapping, breathing/nose, and mouth/throat sounds, respectively. Inquiring trigger sounds with an open question might explain lower percentages in our study. Indeed, subjects might not have taken the time to think of every sound that could be considered as a trigger, and rather focused on the first, and probably main, ones that came to mind. One subject wrote: “so many I can’t even think of more right now but mostly human related noises…”. In Jager et al.^2^, a readymade list of trigger sounds was prepared. Nevertheless, chewing and mouth sounds were the most reported both in this study and in previous ones^2–4,29^.

Besides, differences in category percentages also depend on how sounds are attributed. For instance, we attributed “Swallowing” as a throat sound (focusing on the location of the sound source), whereas Jager et al.^2^, had it in the eating category. Also, we could have eventually assigned some animal sounds to “Repetitive sounds” instead of “Environmental”. However, from subjects’ descriptions it was not clear if it was the repetitive nature of the sound (e.g., continuous dog barking) that was the trigger or not. It would be of interest to have misophonics categorize sounds, instead of researchers. Also, it could be interesting for future research to have a large set of triggers (similar to those self-reported in Fig. 2) rated by misophonics, and then to extract, through factor analysis, empirical categories of misophonic triggers.

### Sound ratings and misophonia

Misophonics had significantly higher ratings than controls for misophonic sounds, while pleasant and unpleasant sounds did not show significant differences (Figs. 3 and 4). As expected, misophonics show high levels of unpleasantness towards specific human generated sounds^2–4^, even at comfortable listening levels. The latter supports previous suggestions that physical characteristics of sounds are less detrimental in sound aversion, rather that it is the previous experiences and associations with triggers that make them unpleasant^1,6^. These results emphasize the differences between misophonia and hyperacusis.

In this study, subjects could decide when the next sound would play. They were given more control than what they usually have over daily situations. Indeed, the context in which sounds are experienced makes the triggers more, or less, uncomfortable. For instance, Edelstein et al.^4^ reported that discomfort was worse when subjects felt they could not escape the situation (e.g. plane trip), which is their main coping mechanism^2,4^. Besides, lack of control of neighborhood sounds makes them more unpleasant, even in the case of non-misophonics^30^. Hence, even in a controlled situation, ratings by misophonics where higher than for controls. This suggests that context is not solely necessary for misophonic aversion, and that underlying emotional and memory associations with sound are sufficient to generate abnormal aversion. Still, as shown recently by Edelstein et al.^7^, context may modulate the severity of such aversion.

Our VAS might measure a mixture of emotion and memory associations related to sounds. For instance, keyboard typing and pen clicking sounds were rated as high even though the sound stimuli were short. This could be explained by two possibilities: a short repetitive stimulus is sufficient to directly induce aversion, and/or it is the memory associations with this type of sound that influenced the rating. These sounds are thought to be annoying because of their repetitive and unpredictable nature^29^, and because of the context in which they are experienced: misophonics do not understand why the originators of the sound do not stop making these rude sounds^4^. In the task, the repetitions (e.g., number of pen clicks) were limited because of the short stimulus (about 2 s), and the context of the sound was not present. Whatever the reasons behind the ratings are, if they are higher than those of controls, it still indicates an abnormal relationship towards sound.

Besides, memory associations of sounds are what are measured with questionnaires. Hence, the advantage of a psychoacoustic assessment is that a mixture of past associations and immediate aversion (lived experience) towards sound is being measured. This measure is also more ecological as it is assessed with actual triggers.

The pathophysiological mechanisms of misophonia are unclear. Trigger sounds have been shown to involve a large network including the anterior insular cortex, hippocampus, and amygdala in misophonic subjects. This network is thought to play a critical role in processing interoceptive signals and emotions^5^. One notes that misophonia has been suggested to be a psychiatric disorder^29^.

Finally, misophonia may have, at least in part, an anthropological and/or sociological origin. For instance, western culture tends to eliminate or “deodorize” body odors^31^. Similarly, western culture tends to eliminate body sounds. It is, for instance, impolite and rude to make sounds when eating, and children are taught to chew with their mouth closed. The sound of chewing may be interpreted as an equivalent of body odor. Like odors, body sounds such as chewing may be used to build the concepts of “oneness” and “otherness”. Besides, like perfume to foul odor, music or other (natural) sounds may be used as a way to mask unwanted sounds^32^ in a social environment. However, the western standards are not necessarily shared by other cultures. Customs regarding eating can be different from one part of the world to another^33,34^. It is unclear whether this cultural aspect may modulate misophonia development, symptoms, and impairment. Misophonia was assessed in Chinese^9^ and American^8^ students using the same misophonia and impairment questionnaires. Average annoyance ratings of misophonic sound categories (e.g., eating, throat, nasal, repetitive sounds) and misophonia prevalence were similar between both studies. However, correlations between misophonia symptoms and functional impairment were lower in the Chinese students than in the American students. It would be of interest to investigate this question of the cultural aspect of misophonia by using our test in different countries.

### The core discriminant sounds as a diagnostic tool for misophonia

With the CDS_Miso_, we selected the most discriminant misophonic triggers, while maintaining sounds from four main subcategories (mouth, breathing/nose, throat, and repetitive sounds), each of which were often reported as triggers in Fig. 2. This suggests that the ratings collected in the task and the method used to select the CDS_Miso_ accurately represent misophonic complaints.

We showed that the ${CDS \, Score }_{Total}$ classified misophonics and controls with 91% accuracy. Each subcategory of the CDS_Miso_ can be used to have a detailed assessment of misophonia and could potentially be used to identify subtypes of misophonia. ${CDS \, Score }_{Mouth}$ had the highest classification accuracy of all subscores (Table 3). This highlights the strong specificity that misophonia has with chewing and eating sounds, which are the most often reported as triggers^2–4,29^.

In Table 4, we attempted to identify potential subtypes of misophonia by using the CDS scores of each subcategory of the CDS_Miso_. Most subjects (55%) had scores from each subcategory above (or equal to) their respective cut-offs. This is not surprising as cut-offs were selected to maximize classification accuracy of our subjects. It would be of interest to test such an approach on another cohort of subjects. Interestingly, cut-off values were computed with rather severe cases of misophonia (high MisoQuest scores). Patterns might emerge in mild to moderate cases of misophonia.

It would also be of interest to correlate CDS scores with precise severity scales of misophonia (e.g., “mild”, “moderate”, or “severe”) to accurately define cut-offs for each severity, for instance using the AMISOS-R^2^.

The CDS_Miso_ were selected based on misophonics identified by the MisoQuest. It would be interesting to verify that correlation found between the CDS Scores and the MisoQuest, is also found with other measures of misophonia, like the MQ^8^ and the AMISOS-R^2^.

### Other limits

Identification of sound seems to influence pleasantness-unpleasantness ratings^35^. In this study, sounds were presented without any text or image identification. Most misophonic sounds are easy to identify with sound alone. However, this might not have been the case for other sounds. Several misophonics mentioned nail sounds as triggers (Fig. 2). In our task, “Fingernails on Chalkboard” was not rated higher by misophonics than controls. This could be because the sound was not recognized and therefore not associated with a visual or tactile perception, or with a past experience. It could be of interest to add information from other sensory modalities, like vision, to the stimuli. Besides, having a subpart of the experiment solely with visual stimuli (e.g., leg rocking) could potentially be used to measure cases of misokinesia.

Cut-offs for each of the CDS scores might have to be reassessed by future studies. Indeed, those shown in Table 3, were computed to optimally classify “clean” subjects. The distribution of CDS scores might not be the same for other cohorts, especially if severity of misophonia is different. This should also be considered if the task is used in a different setting than an online task. It is possible that ratings may slightly differ in a clinical setting than in an online situation. Besides, further validation of the task in a clinical setting, where potential psychiatric and/or audiological comorbidities may be evaluated by the clinician, is important. Furthermore, having a psychoacoustic test only with triggers (e.g., the CDS_Miso_), might change the dynamic of VAS responses, as such cut-offs might also have to be reassessed for this reason.

Future assessment of test–retest reliability of the CDS_Miso_ is important to confirm its viability as a novel assessment tool for misophonia.

The VAS measures the pleasantness-unpleasantness of sound. However, misophonics may experience a variety of emotions that could potentially modulate rated unpleasantness, for instance anger, disgust, stress, or anxiety^4^. It might be of interest to have the CDS_Miso_ evaluated with VAS for each of these.

Finally, gender data was not collected in our study and our French version of the MisoQuest has not been previously validated.

## Conclusions and perspectives

A new assessment tool for misophonia was developed and tested. We further validated a method previously used for hyperacusis assessment^17^, and successfully applied it to another condition (misophonia) and setting (online). We showed that misophonics had higher ratings than controls for misophonic triggers, and not for pleasant or unpleasant sounds, thus further showing that misophonia is specific to certain sounds. The CDS scores can be used to assess misophonia globally, but also to identify potential subcategories of misophonia with a score for each subcategory of the CDS_Miso_.

This psychoacoustic tool, the first that has been developed to assess misophonia, could motivate other studies on conditions where relation to sound is abnormal. For instance, decreased sound tolerance (mainly hyperacusis, but also misophonia) is present in autism^36,37^. A different set of CDS could be more adequate to assess hyperacusis and misophonia in such population.

Using the CDS_Hyp_ and the CDS_Miso_ together in one experiment, with controlled levels, could potentially serve as a novel tool to assess both conditions at once. Tyler et al.^38^ suggested four main types of hyperacusis: loudness, annoyance, fear, and pain. The CDS_Hyp_ probably measure loudness-, and eventually pain- hyperacusis^17^. The CDS_Miso_ most probably measure annoyance hyperacusis, at least annoyance that is specific to the definition of misophonia.

The CDS_Miso_ could potentially be used as an outcome measure over the course of misophonia management. It offers the advantage of directly assessing aversion to triggers at the time they are presented, unlike questionnaire that may not have the proper temporal resolution to assess quick and subtle changes in the lived experience. On the other hand, questionnaires may be more adequate to assess the impact of misophonia on the quality of life, which typically requires extended time to realize if any progress or worsening has been achieved.

## Supplementary Information

Supplementary Information 1.

Supplementary Information 2.

Supplementary Information 3.

Supplementary Information 4.

## Data Availability

All data generated or analyzed during this study are included in this published article’s supplementary information files.
