# Supplementary material for: A psychoacoustic test for misophonia assessment
Source: Sci Rep. 2021 May 26;11:11044. doi: 10.1038/s41598-021-90355-8 (PMC8155015; doi:10.1038/s41598-021-90355-8)
Supplement: Supplementary file 4 — Supplementary Information 4. [file 41598_2021_90355_MOESM4_ESM.docx]

**A psychoacoustic test for misophonia assessment**

*Falco Enzler ^a^, Céline Loriot, Philippe Fournier ^a^ & Arnaud J. Noreña ^a, *^*

^a^ Centre National de la Recherche Scientifique, Aix-Marseille University, 3 Place Victor Hugo, 13003 Marseille, France

**Supplementary Table S1. Frequency of answers to self-report questions.**

|  |  |  | Hearing Issues? | | | Tinnitus? | | | Hyperacusis? | | | Hyperacusis Impact? | | | |
| --- | --- | --- | --- | --- | --- | --- | --- | --- | --- | --- | --- | --- | --- | --- | --- |
|  |  | N | Yes | No | Un. | Yes | No | Un. | Yes | No | Un. | A lot | Mod. | A little | Not at all |
|  | N | 253 | 39 | 190 | 27 | 39 | 196 | 18 | 114 | 114 | 25 | 40 | 40 | 41 | 132 |
| Misophonia? | Yes | 174 | 27 | 126 | 21 | 27 | 134 | 13 | 103 | 54 | 17 | 40 | 38 | 32 | 64 |
|  | No | 45 | 5 | 38 | 2 | 8 | 36 | 1 | 8 | 36 | 1 | 0 | 2 | 5 | 38 |
|  | Un. | 34 | 4 | 26 | 4 | 4 | 26 | 4 | 3 | 24 | 7 | 0 | 0 | 4 | 30 |
| Hearing Issues? | Yes |  |  |  |  | 17 | 15 | 4 | 25 | 8 | 3 | 10 | 7 | 8 | 11 |
|  | No |  |  |  |  | 19 | 164 | 7 | 72 | 101 | 17 | 24 | 24 | 30 | 112 |
|  | Un. |  |  |  |  | 3 | 17 | 7 | 17 | 5 | 5 | 6 | 9 | 3 | 9 |
| Tinnitus? | Yes |  |  |  |  |  |  |  | 26 | 9 | 4 | 8 | 8 | 9 | 14 |
|  | No |  |  |  |  |  |  |  | 78 | 101 | 17 | 26 | 28 | 30 | 112 |
|  | Un. |  |  |  |  |  |  |  | 10 | 4 | 4 | 6 | 4 | 2 | 6 |
| Hyperacusis? | Yes |  |  |  |  |  |  |  |  |  |  | 39 | 37 | 34 | 4 |
|  | No |  |  |  |  |  |  |  |  |  |  | 1 | 2 | 1 | 110 |
|  | Un. |  |  |  |  |  |  |  |  |  |  | 0 | 1 | 6 | 18 |

“Misophonia?”, “Hearing Issues?”, “Tinnitus?”, “Hyperacusis?”, and “Hyperacusis Impact?”, refer to questions 5, 1, 2, 3, and 4, respectively. Un. = Unknown = “I don’t know”. Mod. = Moderately.

**Supplementary Table S2.** **Sounds reported by misophonics as triggers.**

|  | N | % |  | N | % |
| --- | --- | --- | --- | --- | --- |
| **Mouth** | **71** | **91** | **Breathing/Nose** | **35** | **45** |
| Chewing | 42 | 54 | Breathing | 20 | 26 |
| Unspecified | 33 | 42 | Sniffing | 19 | 24 |
| Teeth (brushing, friction, sucking, fork hitting) | 12 | 15 | Snoring | 9 | 12 |
| Tongue click / Licking | 8 | 10 | Whistling nostrils when breathing | 4 | 5 |
| Slurping | 7 | 9 | Other nose sounds (sneezing, snorting, or unspecified) | 3 | 4 |
| Saliva / Spiting | 6 | 8 |  |  |  |
| Nail biting | 5 | 6 |  |  |  |
| Lip smacking | 2 | 3 |  |  |  |
| Yawning | 2 | 3 |  |  |  |
| Specific food (apple bite, popcorn, chips) | 2 | 3 |  |  |  |
| **Throat** | **24** | **31** | **Voices/Talking** | **16** | **21** |
| Swallowing | 11 | 14 | People talking | 7 | 9 |
| Coughing | 7 | 9 | Whispering | 4 | 5 |
| Throat clearing | 6 | 8 | Laughing | 3 | 4 |
| Other throat sounds (burping, gagging, gurgling, or unspecified) | 4 | 5 | Singing | 2 | 3 |
|  |  |  | Crying | 2 | 3 |
|  |  |  | Humming | 2 | 3 |
|  |  |  | Specific words | 1 | 1 |
| **Repetitive Sounds** | **51** | **65** | **Environmental** | **35** | **45** |
| Pen Click | 17 | 22 | Animals (dog barking, rooster, cats, birds) | 11 | 14 |
| Keyboard | 15 | 19 | Miscellaneous * | 11 | 14 |
| Footsteps | 15 | 19 | Traffic noise (car, motorbike, horn, siren) | 8 | 10 |
| Unspecified | 12 | 15 | Impact sounds (door slamming, cymbal) | 5 | 6 |
| Finger/Nail Tapping | 8 | 10 | Friction sounds (clothes, headphones, hands) | 5 | 6 |
| Clock tick | 4 | 5 | Rustling sounds (paper compaction, turning pages, bag of chips) | 5 | 6 |
| Mouse click | 4 | 5 | Water (leak, running, rain) | 4 | 5 |
| Water drop | 3 | 4 | Ventilation | 3 | 4 |
| Finger snapping/cracking | 3 | 4 | Loud sounds | 3 | 4 |
| Other repetitive sounds (music, motor, or words (“um”, “like”)) | 3 | 4 | Neighbor sounds (tv, music) | 2 | 3 |
| **High-Pitched Sounds** | **19** | **24** | **Other** | **14** | **18** |
| Voices / Screams | 7 | 9 | Other nail sounds (clipping, filing, scratching, snapping, and/or unspecified) | 12 | 15 |
| Squeaking / Scratching (breaks, guitar strings, metal scratching, windscreen wipers, door) | 5 | 6 | Other sounds (thumping, leg shaking, or tinnitus) | 3 | 4 |
| Cutlery | 5 | 6 |  |  |  |
| Chalk on blackboard | 2 | 3 |  |  |  |
| Whistling | 2 | 3 |  |  |  |
| Fork/Knife on plate | 2 | 3 |  |  |  |
| Instruments | 1 | 1 |  |  |  |

Sounds were assigned to one of eight main categories (bold). The number and percentage next to each category indicates how many misophonics reported at least one sound within that category as a trigger. Subjects could report more than one sound within each category. “Unspecified” indicates that no specific sound was given, for instance: “any repetitive sound”. Miscellaneous*: plane, church bell, keys, electronic cigarette, bones cracking, construction work, heater, washing hands, writing on table, sliding window, belt buckle, football, clapping, and/or crowded party.

**Supplementary Table S3**. ICC(2,3) detailed results.

| Sound | ICC(2,3) Estimate | F-value | 95% Confidence Intervals |
| --- | --- | --- | --- |
| Birds | 0.962 | 26.63 | 0.953 – 0.970 |
| Blowing Nose | 0.910 | 11.27 | 0.889 – 0.928 |
| Breath Running | 0.896 | 10.32 | 0.866 – 0.919 |
| Chewing 1 | 0.928 | 13.87 | 0.910 – 0.942 |
| Chewing 2 | 0.952 | 20.74 | 0.940 – 0.961 |
| Clapping | 0.915 | 11.98 | 0.895 – 0.932 |
| Cough | 0.853 | 7.17 | 0.814 – 0.884 |
| Distorted Guitar Dissonance | 0.962 | 27.20 | 0.953 – 0.970 |
| Fingernails on Chalkboard | 0.882 | 9.20 | 0.847 – 0.909 |
| Fork Scratch Plate | 0.914 | 11.64 | 0.894 – 0.931 |
| Fountain | 0.904 | 10.66 | 0.881 – 0.923 |
| Gargling | 0.931 | 14.61 | 0.914 – 0.945 |
| Hard Breathing | 0.913 | 11.51 | 0.892 – 0.930 |
| Harp | 0.949 | 19.51 | 0.936 – 0.959 |
| Keyboard | 0.955 | 22.33 | 0.945 – 0.964 |
| Knife Hit Glass | 0.939 | 16.58 | 0.925 – 0.951 |
| Lake | 0.918 | 12.13 | 0.898 – 0.934 |
| Laugh | 0.959 | 25.00 | 0.950 – 0.968 |
| Pen Click | 0.935 | 15.36 | 0.919 – 0.948 |
| Scream | 0.924 | 13.66 | 0.905 – 0.940 |
| Slurping | 0.930 | 14.55 | 0.913 – 0.940 |
| Sniffing | 0.919 | 12.41 | 0.900 – 0.936 |
| Snoring | 0.967 | 30.83 | 0.959 – 0.974 |
| Swallowing | 0.900 | 10.41 | 0.875 – 0.921 |
| Throat Clearing | 0.956 | 22.76 | 0.945 – 0.965 |
| Underwater | 0.912 | 11.48 | 0.891 – 0.929 |
| Vomit | 0.939 | 17.63 | 0.920 – 0.953 |
| Wheezing | 0.910 | 11.27 | 0.889 – 0.928 |

Degrees of freedom 1 and 2, were 243 and 486, respectively.

**Supplementary Dataset S1. Questionnaire Data.** “Hearing Issues?”, “Tinnitus?”, “Hyperacusis?”, “Hyperacusis Impact?”, and “Misophonia?” refer to questions 1 to 5, respectively (c.f. 2.2 Online Task). Those that answered yes to the self-report question on misophonia were asked to list their triggers (“Self-Reported Triggers”).

**Supplementary Dataset S2. All sound ratings results.** Ratings for all 28 sounds for each repetition (n=3) and recruited subject (n=253).

**Supplementary Dataset S3. Normative values for all sounds.** 25% quantile, median, and 75% quantile of the mean ratings of controls with reliable results (n=54) for all 28 sounds.
